# The anti-tumor effect is enhanced by simultaneously targeting VEGF and PROK1 in colorectal cancer

**DOI:** 10.18632/oncotarget.3474

**Published:** 2015-02-28

**Authors:** Takanori Goi, Toshiyuki Nakazawa, Yasuo Hirono, Akio Yamaguchi

**Affiliations:** ^1^ First Department of Surgery, University of Fukui, Japan

**Keywords:** Colorectal cancer, Prokineticin1(PROK1), Vascular endothelial growth factor (VEGF)

## Abstract

Hematogenous metastasis, mainly hepatic metastasis, is a frequent metastatic mode in colorectal cancer involving angiogenic growth factors. Two angiogenic growth factors, in particular, Vascular endothelial growth factor (VEGF) and Prokineticin1(PROK1), are considered to have an important role in hematogenous metastasis of colorectal cancer. Accordingly, we report our findings on the importance of the anti-tumor efffect by inhibiting these two factors in human colorectal cancer.

When the culture fluid of Colorectal cancer cell lines(DLD-1, HCT116, and LoVo) with high levels of VEGF/PROK1 expression was injected subcutaneously into mice, the culture fluid increased subcutaneous angiogenesis. But when both anti-PROK1 and anti-VEGF antibodies were present in the culture fluid, the length and size of the blood vessels were reduced compared with those seen in the fluid-only, anti-PROK1, and anti-VEGF controls. Also, tumor masses were produced in mice by subcutaneously embedding colorectal cancer cells with high levels VEGF/PROK1 expression. When both anti-PROK1 and anti-VEGF antibodies were simultaneously applied, tumor formation and peritumoral angiogenesis were strongly suppressed, compared with when either anti-PROK1 antibody or anti-VEGF antibody was applied alone.

Simultaneous targeting of both angiogenic growth factors(VEGF/PROK1) may prove more useful in colorectal cancer.

## INTRODUCTION

Colorectal cancer is a highly prevalent malignancy in the Western World, Japan, and other countries [1-3]. The prognosis of colorectal cancer at an early stage is favorable. Thanks to the recent progression of anticancer agents and molecular target therapy, prognoses are generally improved, but the prognosis of unresectable, advanced colorectal cancer is not yet satisfactory. While there are various metastatic modes in colorectal cancer such as lymph node, peritoneal and hematogenous metastases, a majority of patients face poor prognosis due to hepatic and other hematogenous metastases [4-6]. Therefore, countermeasures against hematogenous metastasis will be most important for improving the prognoses of these cancer patients.

The possible mechanism of hematogenous metastasis of colorectal cancer is as follows: dissociation from the primary lesion, disintegration of the basement membrane, movement into the interstitium, invasion into the vascular channel, and colonization of target organs during the final stage [7, 8]. In recent years, molecular biological investigations have been undertaken to study the metastasis of various tumors, and the involvement of a number of factors has been confirmed [9-12]. Various molecular target drugs have been used and listed in NCCN's Guidelines for the Treatment of Colorectal Cancer [13]. In particular, there are drugs that target molecules related to the angiogenic growth factor:vascular endothelial growth factor (VEGF) (anti-VEGF antibody and anti-VEGF receptor antibody), a drug that targets the intracellular signaling mechanism-related EGF receptor (anti-EGFR antibody), and a multikinase inhibitor that targets receptor tyrosine kinases [14-18].

VEGF is known to act on the pericellular interstitium to obtain oxygen and energy [19-21]. A report showed that intensifying VEGF expression activated proliferation of liver metastatic lesions in mice [22]. Furthermore, as suppression of VEGF activity inhibits the proliferation of cancer cells, the prognosis for patients with unresectable colorectal cancer can be improved [23]. Close relationships between VEGF and hematogenous metastasis were reported for other malignant tumors such as lung, breast and renal cancer [24-27]. Meanwhile, our study involving a colorectal cancer cell line with low levels of Prokineticin1(PROK1) expression showed that angiogenesis, tumor proliferation, and hematogenous metastasis occurred at high rates in the surrounding tissues when the PROK1 gene was introduced [28-30]. Intensification of PROK1 expression was also observed in advanced-stage gastric and small intestine cancer [31]. Other institutions reported the relationship between PROK1 expression and malignancy in prostate cancer, neuroblastoma, and pancreatic cancer [32-35].

As very few studies have been conducted to determine the effects of various angiogenic growth factors, we decided to examine the interactions between VEGF and PROK1, which are two important angiogenic growth factors for hematogenous metastasis in colorectal cancer.

## RESULTS

### PROK1/VEGF expression in colorectal cancer cell lines

**Figure 1:**
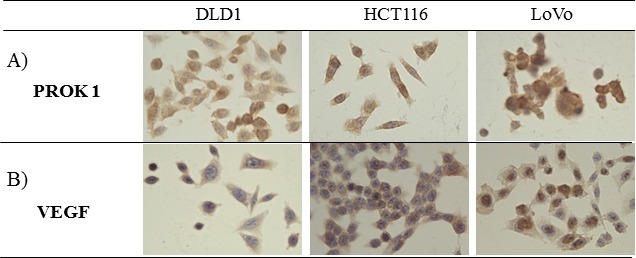
The expression of PROK1 and VEGF protein in colon cancer cell lines(DLD-1, HCT116, LoVo) by immunohistochemical staining with anti-PROK1 mAb A) PROK1 protein expression was detected in all colon cancer cell lines. B) VEGF protein expression was detected in all colon cancer cell lines.

Immunohistochemical staining showed PROK1/VEGF expression in the human colorectal cancer cell lines LoVo, HCT116, and DLD-1 (Fig. 1).

### Angiogenesis in the subcutaneous tissue of mice after injection of colorectal cancer cell culture fluid containing anti-PROK1 antibody and anti-VEGF antibody

**Figure 2:**
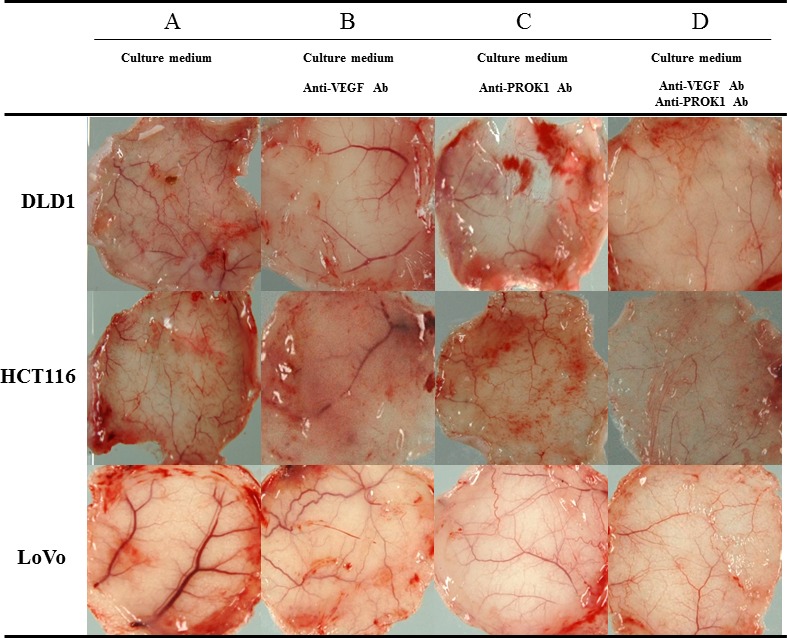
Representative photographs of angiogenesis in mice in response to colorectal cancer cell fluid(colorectal cancer cell lines:DLD-1, HCT116, HT29) A) Culture fluid alone, B) culture fluid plus the anti-VEGF mAb, C) culture fluid plus the anti- PROK1 mAb, D) culture fluid plus the anti-VEGF mAb and anti-PROK1 mAb.

Subcutaneous angiogenesis after injection of colorectal cancer cell culture fluid was assessed in mice. The length and diameter of blood vessels were suppressed when anti-VEGF antibody or anti-PROK1 antibody was added to the culture fluid, compared with when culture fluid contained no additional antibody. When both anti-VEGF antibody and anti-PROK1 antibody were present, the blood vessels were further reduced in length and diameter (Fig. 2).

### CD31 expression in mouse subcutaneous tissue after injection of colorectal cancer cell culture fluid containing anti-PROK1 antibody and anti-VEGF antibody

**Figure 3:**
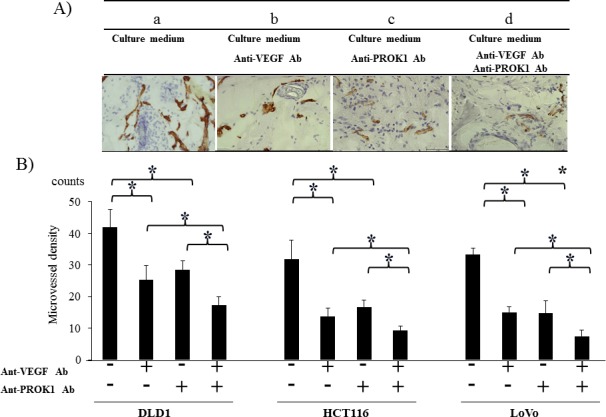
Investigation of CD31 immunohistochemical staining in mice in response to colorectal cancer cell fluid(colorectal cancer cell lines:DLD-1, HCT116, HT29) A) Representative photographs of anti-CD31Ab stained cells by performing immunohistochemical staining. a) HCT116 culture fluid alone, b) HCT116 culture fluid plus the anti-VEGF mAb, c) HCT116 culture fluid plus the anti-PROK1 mAb, d) HCT116 culture fluid plus the anti-VEGF mAb and anti-PROK1 mAb. B) The numbers of positively CD31 stained cells in mice in response colorectal cancer cell fluid(DLD-1, HCT116, HT29). Data represent means ± SEM. (*student t-test p<0.01).

Immunohistochemical staining was conducted using an anti-CD31 monoclonal antibody on the same mouse subcutaneous tissue, and the positively stained cells were counted (Fig. 3A). There were 42 CD31-positive cells per field-of-view when DLD-1 culture fluid was used alone, 27, 25 per field-of-view in the presence of anti-PROK1 antibody or anti-VEGF antibody, and 17 per field-of-view in the presence of both antibodies. When HCT116 culture fluid containing no additional antibodies was injected, 31 positive cells were observed per field-of-view, 17,13 were observed per field-of-view in the presence of either anti-PROK1 antibody or anti-VEGF antibody, and 7 were observed in the presence of both antibodies. When LoVo culture fluid containing no additional antibodies was injected, 32 positive cells were observed per field-of-view. Sixteen cells were observed per-field-of view in the presence of either anti-PROK1 antibody or anti-VEGF antibody, and 5 were observed in the presence of both antibodies (Fig. 3B). For all of the cell lines, the number of stained cells was significantly fewer in the presence of both anti-VEGF and anti-PROK1 antibodies.

### Suppression of tumor formation by colorectal cancer cells after application of anti-PROK1 antibody and anti-VEGF antibody

**Figure 4:**
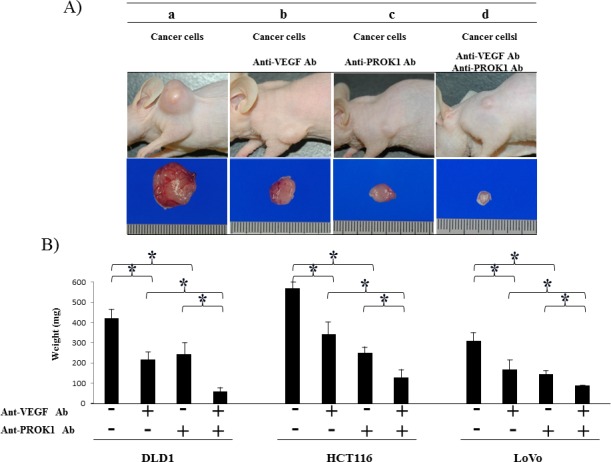
Suppression of mouse colorectal cancer cell line tumor formation by the anti-PROK1mAb and anti-VEGF mAb SHO nude mice were subcutaneously injected in the right armpit region with 1.0 × 10^6^ colorectal cancer cells(DLD-1, HCT116, HT29) and the anti-PROK1 mAb and anti-PROK1 mAb. A) Representative photographs of tumor formation in mice in response to colorectal cancer cells(HCT116). Three weeks later, the tumor was photographed. a) Culture fluid alone, b) culture fluid plus the anti-VEGF mAb, c) culture fluid plus the anti-PROK1 mAb, d) culture fluid plus the anti-VEGF mAb and anti-PROK1 mAb. B) Three weeks later, the tumor was resected and weighted. The measurement of subcutaneous tumor weight. Data represent means ± SEM. (*student t-test p<0.05).

Cultured HCT116 cells were subcutaneously injected in mice (Fig. 4A). The size of the resulting tumor was 550 mg when no antibodies were added 340 mg in the presence of anti-VEGF antibody, 230 mg in the presence of anti-PROK1 antibody, and 110 mg in the presence of both anti-VEGF and anti-PROK1 antibodies. When DLD-1 cells were injected without additional antibodies, the size of the subcutaneous tumor was 410 mg. It was 210 mg in the presence of anti-VEGF antibody, 230 mg in the presence of anti-PROK1 antibody, and 50 mg in the presence of both antibodies. When LoVo cells were injected, the tumor was 290 mg. It was 140 mg in the presence of anti-VEGF antibody, 120 mg in the presence of anti-PROK1 antibody, and 90 mg in the presence of both anti-VEGF and anti-PROK1 antibodies (Fig. 4B). For all of the cell lines, tumor formation was significantly suppressed in the presence of both anti-VEGF antibody and anti-PROK1 antibody, compared with when only anti-VEGF or anti-PROK1 was present.

### CD31 expression in colorectal cancer cell lines with the addition of anti-PROK1 antibody and anti-VEGF antibody

**Figure 5:**
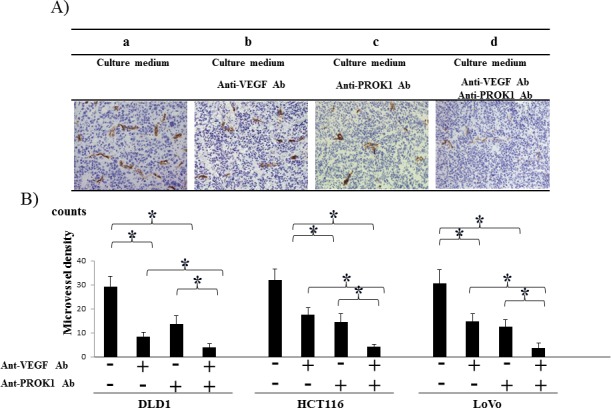
Investigation of CD31 immunohistochemical staining of subcutaneous tumor formation by the anti-PROK1Ab and anti-VEGF mAb A) Representative photographs of anti-CD31Ab stained cells by performing immunohistochemical staining. a) HCT116 alone, b) HCT116 plus the anti-VEGF mAb. c) HCT116 plus the anti- PROK1 mAb, d) plus HCT116 plus the anti-VEGF mAb and anti-PROK1 mAb. B) The numbers of positively CD31 stained cells in subcutaneous tumors(DLD-1, HCT116, HT29). Data represent means ± SEM. (*student t-test p<0.05).

Immunohistochemical staining was conducted using anti-CD31 monoclonal antibody in the same mouse tumors (Fig. 5A), and the positively stained cells were counted (Fig. 5B). The number of positive cells in the DLD-1 tumors was 29 per field-of-view, 13, 9 per field of view in the presence of anti-PROK1 antibody or anti-VEGF antibody, and 3 in the presence of both antibodies. In the HCT116 tumors, 32 positive cells per field-of-view were observed with no additional antibodies. Approximately 14, 18 cells were observed in the presence of either anti-PROK1 antibody or anti-VEGF antibody, and 4 were observed in the presence of both antibodies. In the LoVo tumors, there were 29 positive cells per field-of-view with no additional antibodies, 13, 14 in the presence of either anti-PROK1 antibody or anti-VEGF antibody, and 3 in the presence of both antibodies. In all of the cell lines, the number of stained cells was the smallest when anti-VEGF and anti-PROK1 antibodies were present simultaneously.

## DISCUSSION

Molecular biological investigations have been undertaken for various malignant tumors, and angiogenic growth factors are known as important prognosis-determining genes for patients whose colorectal cancer has undergone hematogenous metastasis, in particular [38-41].

Among the molecule-targeting drugs for unresectable advanced recurrent colorectal cancer that are listed in the current NCCN's Guidelines for Treatment of Colorectal Cancer [13], many of the angiogenesis-related drugs target VEGF-associated molecules. Three types are listed as efficacious drugs. Bevacizumab has a VEGF-neutralizing effect and a vascular normalization which improves the delivery and effectiveness of chemotherapeutics [14, 21, 42]. Ziv-Aflibercept is a receptor-antibody complex consisting of VEGF-binding portions from the extracellular domains of VEGF Receptors 1 and 2 and fused to human immunoglobulin IgG1Fc [18]. Regorafenib targets VEGFR1-3, TIE2 [17], and other receptor tyrosine kinases. VEGF is thought to act on the interstitium around the cancer cells and induce angiogenesis, thus leading to proliferation and metastasis of cancer cells. According to a report, VEGF and hematogenous metastasis are closely related in lung, breast, and renal cancer [24-26]. And PROK1 is thought to work as an angiogenic growth factor in colorectal cancer [27, 28, 30] and to be involved in autocrine mechanism-induced infiltration of cancer cells [43]. Also, PROK1 levels were reported to be related to the degree of malignancy in gastric cancer, small intestine cancer, pancreatic cancer, neuroblastoma, and prostatic cancer [30-34]. Recently we found that PROK1 protein was observed in the culture fluids of all colorectal cancer cell lines: DLD-1, HCT116, and LoVo [37].

Both VEGF and PROK1 have been confirmed as significant factors in colorectal cancer, though expression of the two factors has not been investigated in this context. We undertook the present study to determine whether these two factors could be possible therapeutic targets. According to our results, while angiogenesis and tumor growth were suppressed by using either factor, the suppressive effect of using both factors simultaneously was greater. (Anti-VEGF Ab in the present experiment is different from Bevacizumab). Since we have no significance using two antibodies together, further clinical studies are necessary.

In terms of mechanism of action, dimers are formed following the binding of VEGF to its receptors, VEGFR-1 and VEGFR-2, on the surface of vascular endothelial cells [44]. Autophosphorylation occurs and the MAPK cascade starts, transmitting cell growth signals into the nucleus [45]. As a result, vascular growth occurs, and cancer cells digest food and oxygen for growth. G protein-coupled receptors (PROKR1 and PROKR2) are the receptors for PROK1 and are expressed in the endothelial cells and cancer cells themselves [46, 47]. Intracellular calcium kinetics, phosphorylation of p44/p42MAP, and serine-threonine kinase Akt are involved downstream of the receptor [48], playing an important role in vascular growth, tumor growth, anti-apoptosis function, differentiation, and other cell kinetic behaviors [49].

To summarize, VEGF and PROK1 are thought to exert important functions via individual receptors to transmit different intracellular signals. Our results suggest that angiogenesis and the tumor growth rate are significantly suppressed in the presence of both anti-VEGF and anti-PROK1 antibodies together, compared with in the presence of only one or the other. Therefore, dual application of both antibodies may be developed into an effective cancer therapy.

## MATERIALS AND METHODS

### Cell culture

The human colon cancer cell lines, DLD-1, HCT116 and LoVo(obtained from the European Collection of Cell Cultures in 2013, Culture Collections of Public Health England, UK) were maintained by our laboratory and cultured in RPMI1640 medium supplemented with 10% fetal bovine serum(FBS), 100 U/mL streptomycin and 100 U/mL penicillin (Gibco/Invitrogen, USA) at 37°C in 5% CO2 [36].

### Cell culture fluid

Each cell line was passaged at 60% confluence in a 60-mm culture dish, and cultured in RPMI1640 containing 10% FBS for 3 days. The culture fluid was collected after culture of the cell lines.

### Antibody(Ab)

The primary antibodies used were anti-VEGF (Santa Cruz Biotechnology, USA), anti-CD31 (DAKO, Danmark), and anti-PROK1(established by our department) [37].

### Detection of vascularization with Dorsal air sac method

A Millipore chamber(Millipore; diameter, 10mm: filter pore size, 0.45μm) was filled with culture medium plus Ab or normal mouse IgG was implanted subcutaneous tissue into the dorsal side of six-week-old female SHO nude mice (Charles river, Japan). At 7 days after implantation, a incision was made in the skin on the dorsal side. The chamber-contacting region was photographed.

### Tumor formation and microvessel counting in nude mice

Six-week-old female SHO nude mice(Charles river, Japan) were subcutaneously injected in the armpit region with 1.0 × 10^6^ cells in 0.1 mL of matrix gel(BD Biosciences, USA). Four groups of mice were tested. Group A was injected with non-stimulated colon cancer cells (DLD-1, HCT116 and LoVo) and normal mouse IgG. Group B was injected with colon cancer cells and the anti-VEGF mAb (5μg). Group C was injected with colon cancer cells and the anti-PROK1 mAb (5μg). Group D was injected with colon cancer cells and anti-VEGF mAb(5μg) and the anti-PROK1 mAb(5μg). The tumor size was measured every 3 days with calipers. The tumor volume was calculated with the formula: (L × W^2^)/2, where L is the length and W is the width of the tumor [28]. After 21 days, the tumor was resected, photographed, and weighted.

### Immunohistochemical study

Tumors and subcutaneous tisseues were resected and embedded in OCT compound (Sakura Finetechnical, Japan). Four-μm-thick sections were analyzed for the expression of CD31 protein by the ChemMate method using the EnVision system(DAKO). For vessel counting, one field magnified 200-fold in each of five vascularized areas was counted, and average counts were recorded.

### Statistical analysis

Statistical significance was performed by the student t-test using Stat Mate IV(ATMS Co., Ltd., Japan). Data are given as mean ± SEM. Differences were considered significant at *P* values less than .05.
